# Nutcracker Phenomenon with Menorrhagia in a Woman with Marfan Syndrome

**DOI:** 10.3400/avd.cr.19-00055

**Published:** 2021-03-25

**Authors:** Kayo Sugiyama, Toshiki Fujiyoshi, Nobusato Koizumi, Hitoshi Ogino

**Affiliations:** 1 Department of Cardiovascular Surgery, Tokyo Medical University Hospital, Tokyo, Japan; 2 Department of Cardiac Surgery, Aichi Medical University Hospital, Nagakute, Aichi, Japan

**Keywords:** nutcracker phenomenon, pelvic congestion syndrome, Marfan syndrome

## Abstract

Nutcracker phenomenon (NCP) refers to left renal vein compression at the superior mesenteric artery origin involving hematuria and dysuria due to the compression of the renal venous return and pelvic congestion syndrome caused by the compression of the gonadal venous return. A leptosomatic woman (body mass index, 19 kg/m^2^) presented with NCP and Marfan syndrome accompanied by severe menorrhagia. Vascular ultrasonography revealed reversed flow in the left ovarian vein. Preoperative computed tomography revealed a sharp aortomesenteric angle and short aortomesenteric distance. After controlling her menstrual period via oral contraception, she underwent valve-sparing surgery for aortic root dilation, which spontaneously subsided the menorrhagia.

## Introduction

Nutcracker phenomenon (NCP) refers to the vascular entrapment of the left renal vein between the aorta and superior mesenteric artery. The typical features of NCP involve underlying compression of the left renal vein, which results in hematuria and dysuria.^1,2)^ NCP also causes pelvic congestion syndrome with reversed flow in the left gonadal vein, resulting in chronic pelvic pain and dysmenorrhea. However, metrorrhagia and menorrhagia rarely occur.^3–5)^ A low body mass index is associated with the occurrence of NCP as the aortomesenteric angle can be sharp, and the aortomesenteric distance can be short.^6–9)^ Although there have been no reports on the relationship between Marfan syndrome (MFS) and NCP, NCP should be considered in leptosomatic patients suffering from pelvic congestion syndrome.

## Case Report

**Figure 1:**
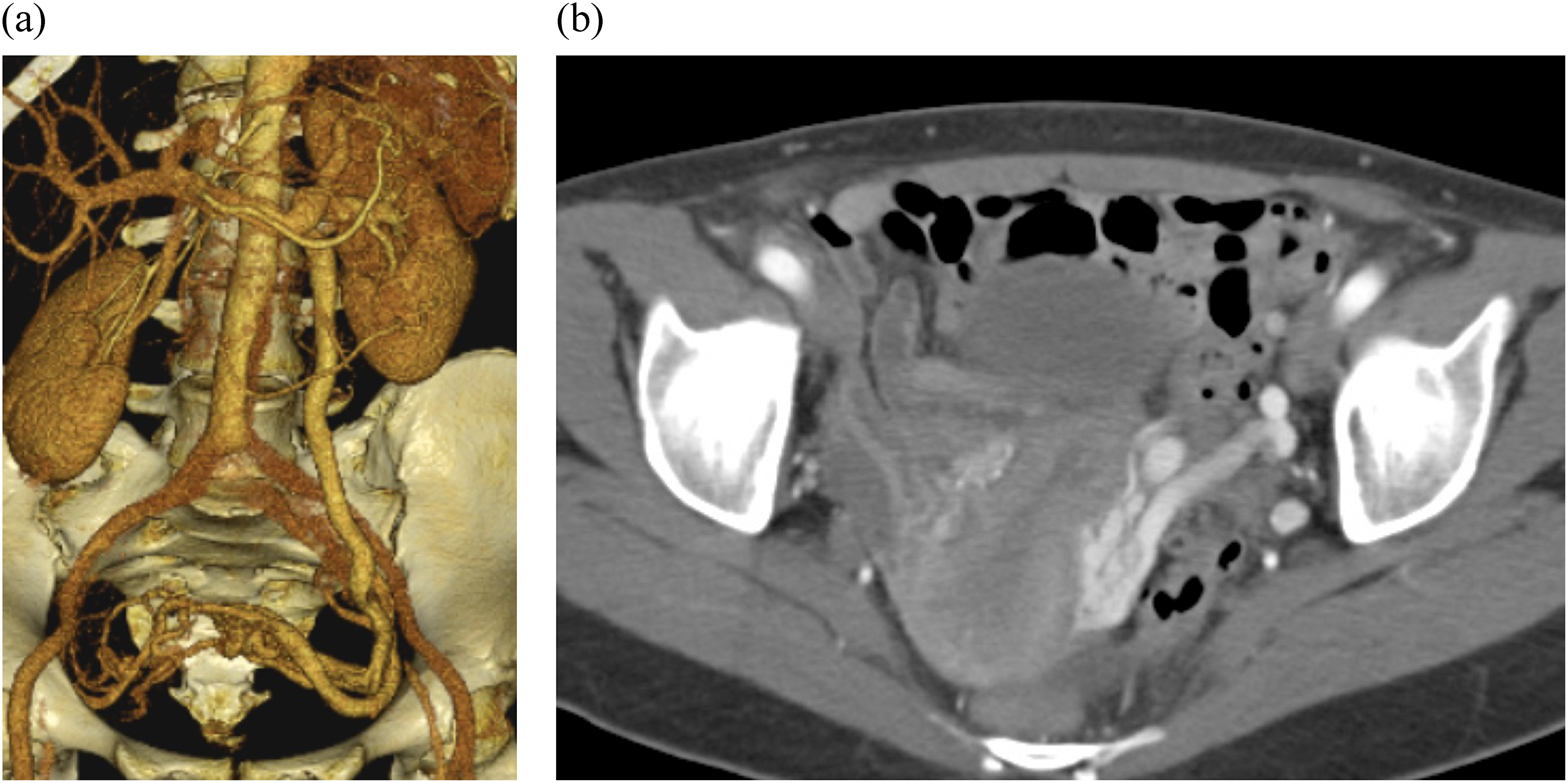
Fig. 1 Preoperative computed tomography scan images showing compression of the left renal vein with dilation of the left gonadal vein.

**Figure 2:**
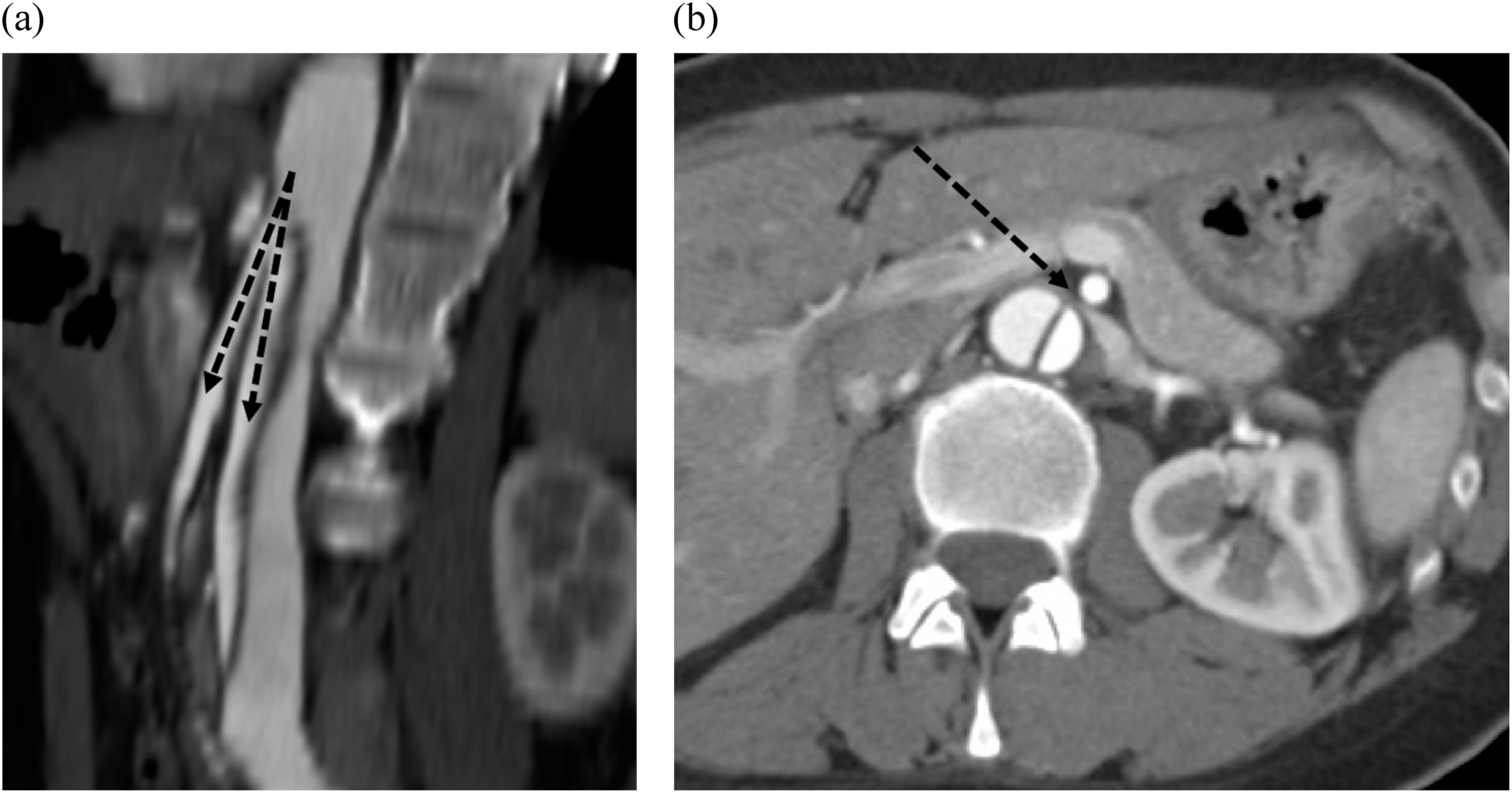
Fig. 2 (**a**) A preoperative computed tomographic scan revealing the sharp aortomesenteric angle (dotted arrows). (**b**) A preoperative computed tomographic scan revealing the short aortomesenteric distance (dotted arrow).

A 45-year-old woman with MFS (height, 167 cm; weight, 55 kg; body mass index, 19 kg/m^2^) was admitted to our institute to undergo aortic valve-sparing surgery for aortic root dilation. She underwent a type B aortic dissection 5 years previously, and her father and brother had also been diagnosed with MFS. She complained of severe menorrhagia but denied any other symptoms relating to chronic venous insufficiency. Her laboratory test results were normal, except for low-grade anemia (hemoglobin, 9 mg/dL). An echocardiography revealed aortic root dilation, moderate aortic valve regurgitation, and normal cardiac function. A computed tomography (CT) scan revealed dilation of the aortic root (diameter, 5 cm), chronic type B aortic dissection, and dilation of the left ovarian vein (**Figs. 1a** and **1b**). The angle between the abdominal aorta and superior mesenteric artery (aortomesenteric angle) was found to be extremely sharp (10°) (**Fig. 2a**), and the distance between the abdominal aorta and superior mesenteric artery at the level of the left renal vein (aortomesenteric distance) was exceedingly short (3 mm) (**Fig. 2b**). Vascular ultrasonography revealed reversed flow of the left ovarian vein. Other relevant clinical examinations for severe menorrhagia and anemia revealed no other remarkable findings; therefore, the etiology of menorrhagia was inferred to be pelvic congestion syndrome due to NCP.

**Figure 3:**
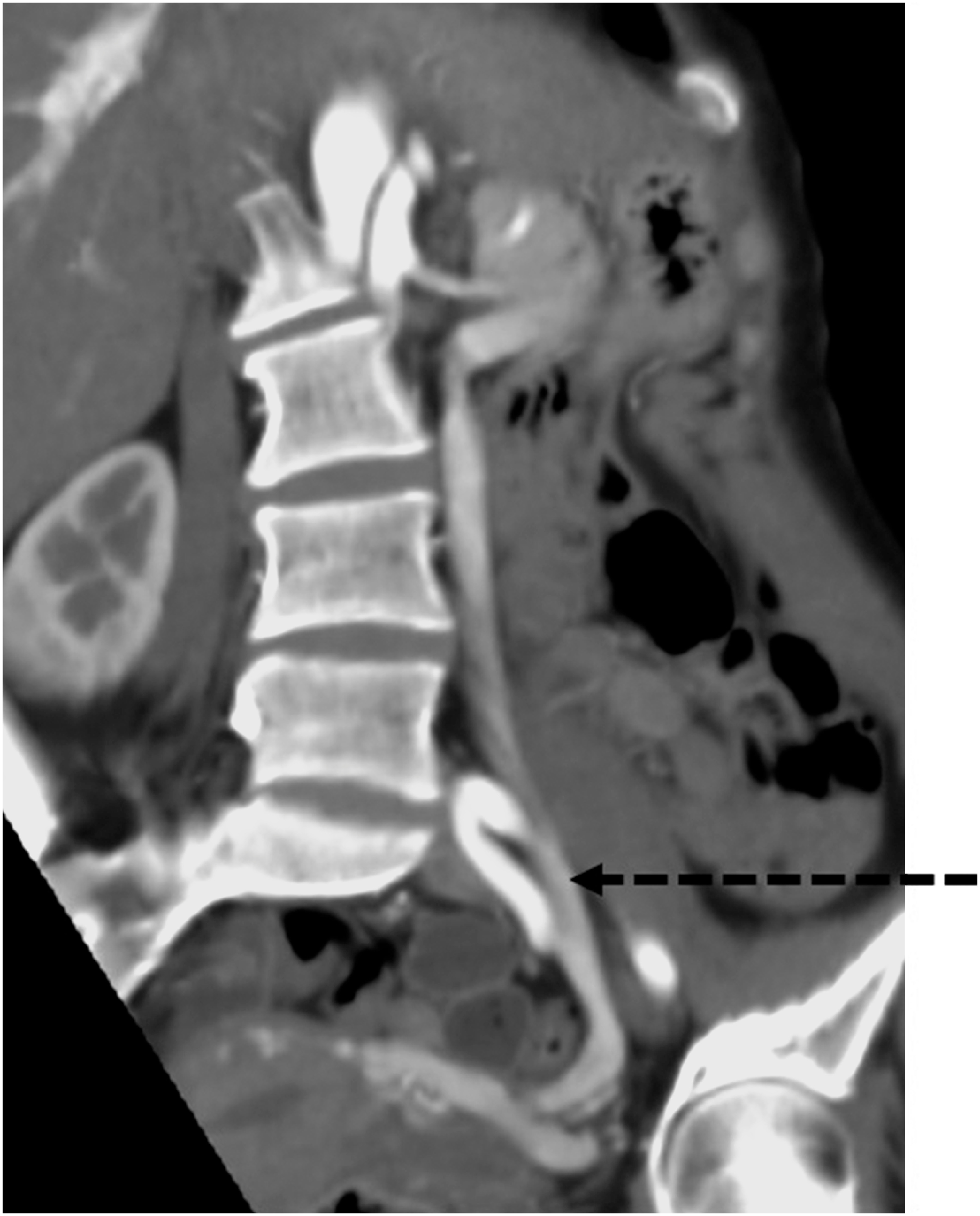
Fig. 3 A postoperative computed tomographic scan revealing mild dilation of the left gonadal vein (dotted arrow).

Preoperatively, gynecologists were consulted concerning control of her menstrual period. After the administration of oral contraceptive medication, we chose to perform aortic valve-sparing surgery for the following reasons. First, this type of surgery does not require the administration of anticoagulant therapy, and our patient was a young, fertile woman. Second, the shape of the aortic valve appeared suitable for valve-sparing surgery. Third, at our institution, previous results involving this type of surgery have been acceptable. Moreover, we planned to provide additional treatment for NCP had her menorrhagia symptoms persisted postoperatively. Valve-sparing surgery was successfully performed, and because anticoagulant therapy was no longer needed, she did not experience any genital bleeding. A postoperative CT scan revealed mild dilation of the left ovarian vein (**Fig. 3**); however, her symptoms of severe menorrhagia spontaneously subsided. At 3 years post-discharge, our patient continues to do well, with no reports of any major adverse cardiac events or symptoms related to pelvic congestion syndrome.

## Discussion

NCP expressions range from asymptomatic microscopic hematuria to severe metrorrhagia requiring blood transfusion. NCP also causes pelvic congestion syndrome with reversed flow in the left gonadal vein, resulting in chronic pelvic pain and dysmenorrhea. However, metrorrhagia and menorrhagia rarely occur.^3–5)^ The progression of this syndrome is reportedly related to the sharpness of the aortomesenteric angle and the shortness of the aortomesenteric distance.^2)^ Similarly, a low body mass index has been reported to be associated with NCP,^6–9)^ and dieting and eating disorders have also been previously described as risk factors.^6–9)^ Our findings revealed that this patient’s body mass index was 19 kg/m^2^, the aortomesenteric angle was 10°, and the aortomesenteric distance was 3 mm. Although there have been no studies on the relationship between MFS and NCP, NCP should be considered in leptosomatic patients with MFS who complain of symptoms suggestive of pelvic congestion syndrome. Further reports on the relationship between MFS and NCP are warranted.

We had planned to provide additional treatment for NCP if menorrhagia had persisted after cardiac surgery. Valve-sparing surgery was chosen in this case as it does not require anticoagulant therapy administration. Fortunately, the symptoms spontaneously subsided, and our patient has been free from severe menorrhagia for 3 years. However, a careful observation is required. Although the exact reason why the bleeding stopped is unknown, it may have been helpful that valve-sparing aortic root repair does not require anticoagulant therapy. Furthermore, dieting and eating disorders have also been previously described as risk factors of NCP.^5,8)^ It has been reported that conservative management with emphasis on weight gain increases the retroperitoneal adipose tissue, leading to a change in the positioning of the left kidney with reduction of tension on the left renal vein. It is speculated that postoperative improvement in nutritional status may have had an effect.^4,10)^ Alternatively, the development of collaterals in the pelvis might have been effective.^4,10)^ No reports have described the rationale for cardiac surgery in patients with pelvic congestion syndrome. However, to prevent genital bleeding, menstruation control via oral contraception is recommended prior to cardiac surgery.

## Conclusion

We described a case of MFS with NCP-related severe menorrhagia. NCP should be considered in leptosomatic patients with pelvic congestion syndrome.
